# The effect of graded activity and pain education (GAPE): an early post-surgical rehabilitation programme after lumbar spinal fusion—study protocol for a randomized controlled trial

**DOI:** 10.1186/s13063-020-04719-y

**Published:** 2020-09-15

**Authors:** Heidi Tegner, Bente Appel Esbensen, Marius Henriksen, Rachid Bech-Azeddine, Mari Lundberg, Louise Nielsen, Nanna Rolving

**Affiliations:** 1 Department of Occupational Therapy and Physiotherapy, Rigshospitalet, Valdemar Hansens Vej 13, 2600 Glostrup, Denmark; 2 Centre for Rheumatology and Spine Diseases, Rigshospitalet, Valdemar Hansens Vej 13, 2600 Glostrup, Denmark; 3 The Parker Institute, Copenhagen University Hospital, Bispebjerg/Frederiksberg, Nordre Fasanvej 57, 2000 Frederiksberg, Denmark; 4 Department of Health and Rehabilitation, University of Gothenburg, Box 455, 405 30 Gothenburg, Sweden; 5 DEFACTUM, Corporate Quality, Central Denmark Region, P.P. Oerumsgade 11, 8000 Aarhus C, Denmark

**Keywords:** Low back pain, Surgery, Rehabilitation, Behaviour

## Abstract

**Background:**

Patients with chronic low back pain undergoing lumbar spinal fusion (LSF) are physically inactive and thereby at risk of poor health. Barriers to being physically active need to be acknowledged in post-surgical rehabilitation. The primary objective of this randomized controlled trial (RCT) is to examine the effect of an early active intervention consisting of graded activity and pain education (GAPE) on sedentary behaviour in a population of patients undergoing LSF. The secondary objective is to examine the effect of GAPE on disability, pain, fear of movement, self-efficacy for exercise, and health-related quality of life.

**Methods:**

The study is an RCT planned to include 144 patients undergoing LSF at 1–2 levels for low back pain caused by degeneration of the lumbar spine. The patients will be randomly assigned to receive either usual care or usual care plus GAPE. GAPE consists of nine individual physiotherapist-guided sessions over a 10-week period. The overall purpose is to reduce sedentary behaviour, by educating the patient about pain and, based on a cognitive behavioural perspective, gradually strengthen the patient’s self-efficacy to be physically active and reduce fear of movement. The physiotherapist will plan the intervention in collaboration with the patient. Based on a semi-structured interview and observations of the patient in their home, they will set individually functional goals. The primary outcome will be a reduction in sedentary behaviour, measured by an accelerometer at baseline (pre-surgery) and at 3 and 12 months post-surgery. Secondary outcomes will include disability, pain, fear of movement, self-efficacy for exercise, and quality of life. Secondary outcome data will be collected at baseline (pre-surgery) and at 3, 6 and 12 months post-surgery.

**Discussion:**

We hypothesize that, compared with the “usual care group”, GAPE will primarily lead to a significant reduction in sedentary behaviour, and secondarily a reduction in disability, pain intensity, and fear of movement; further, it will increase the patient’s self-efficacy for exercise and quality of life.

**Trial registration:**

www.clinicaltrials.gov NCT04103970, Registered on 24 September 2019

## Background

Over half a billion people worldwide report low back pain (LBP), which is the leading cause of disability at all income levels and age groups [1, 2]. For the majority of people LBP has a natural benign course, but for a small percentage, it turns into a chronic condition with significant levels of life disruption, healthcare costs, economic losses, and even premature death [1]. Lumbar spinal fusion (LSF) is a widely adopted surgical procedure for the treatment of persistent LBP, with the aim of relieving pain and thereby increase functional ability [3, 4]. Over recent decades, a substantially increasing number of patients with chronic low back pain (CLBP) undergo LSF in the Western world [4]. In the USA, the number of patients undergoing LSF increased by 62% from 2004 to 2015 and in the UK by 63% from 2005 to 2015 [3, 4].

Low physical activity is a global health problem and may add a greater risk for a poor surgical outcome for patients undergoing LSF [5]. A cross-sectional study found that 83% of patients scheduled for LSF did not adhere to the World Health Organization recommendations regarding physical activity, i.e. 30 min per day [5]. Further, this seems to be unchanged 6 months after LSF [6], indicating that these patients may be at risk of poor health and lifestyle diseases due to insufficient physical activity [7]. Physical activity should hence be incorporated in all post-surgical rehabilitation programmes to achieve a healthy physical activity behaviour.

Fear of movement is one barrier identified in as many as 70% of patients planned for spinal surgery [8]. Fear of movement, avoidance coping, negative affect, and depression postoperatively are associated with persistent pain and reduced function after spine surgery up to 3 years postoperatively [9–14]. Individual differences in pain-related coping strategies after LSF also seem to have an influence on the patient’s sedentary behaviour postoperative [12].

There is growing evidence to suggest that, to increase physical activity and function in this group of patients, post-surgical rehabilitation should start early, and the content of the rehabilitation should be based on a bio-psycho-social approach [5, 15–17]. A systematic review by Greenwood et al. found that “complex interventions”, consisting of exercise and cognitive behavioural therapy, offer short-term and long-term functional benefits for patients following LSF [18]. Archer et al. confirmed that an early intervention using a cognitive behavioural approach performed by a physiotherapist decreased fear of movement, increased self-efficacy, and improved patient-reported and performance-based outcomes in patients 6 months after lumbar spine surgery [19]. Research in the field of rehabilitation after LSF calls for high-quality research, which includes interventions that incorporate the patients’ context, experiences, and thoughts to a greater extent in the clinical decision making [18, 19].

In this suggested randomized controlled trial (RCT), we will investigate the effect of a bio-psycho-social approach consisting of graded activity and pain education. In short, we will call it GAPE. We will include the patient’s environment and perspectives in the intervention through home visits, explorative interviews, and specific, individual goals set by the patient in collaboration with the physiotherapist.

Graded activity is an exercise paradigm which takes a behavioural perspective using the principles of operant conditioning [20]. The purpose of operant conditioning are to modify negative pain behaviours and break the fear-avoidance cycle and thereby increase the patient’s physical activity and functioning [21]. Several studies have investigated the effect of pain education in patients with CLBP and found it to be efficient in terms of less pain and increased functional level [22, 23]. Furthermore, a combination of exercise and pain education for patients with CLBP has showed promising results [24–26]. A combination of graded activity and pain education (GAPE) seems to be a suitable supplement to early post-surgical rehabilitation for patients with LSF, given that behavioural, cognitive, physical, and contextual factors will be addressed in one intervention.

### Objective and hypothesis

The primary objective of this RCT is to examine the effect of an early active post-surgical intervention consisting of GAPE on sedentary behaviour in a population of patients undergoing LSF. The secondary objectives are to examine the effect of GAPE on disability, pain, fear of movement, self-efficacy for exercise, and health-related quality of life.

We hypothesize that, compared to usual care, GAPE will primarily lead to a significant reduction in sedentary behaviour and, secondly, reduced disability, pain intensity, and fear of movement; further, it will increase the patient’s self-efficacy for exercise and quality of life.

## Methods/design

The study is a parallel-group RCT with 3, 6, and 12 months of follow-up. Patients will be randomized 1–2 days post-surgery (1:1) to either usual care or GAPE in addition to usual care (see the “Randomization” section).

**Fig. 1:**
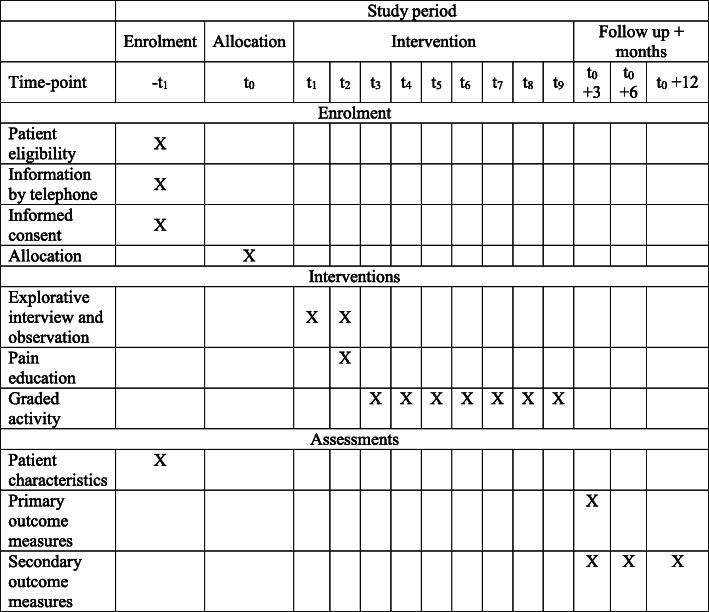
SPIRIT diagram for trial stages of enrolment, intervention, and assessment

The trial is reported according to the Standard Protocol Items: Recommendations for Interventional Trials (SPIRIT) Statement [27] (see Additional file 1 and Fig. 1).

### Study setting

GAPE will be provided by telephone, in the patient’s home, and in the training facilities of the Department of Occupational Therapy and Physiotherapy, Rigshospitalet, Glostrup. At the hospital, the explorative interview will be carried out when the patient is still hospitalized at the Centre for Rheumatology and Spine Diseases.

### Patients

#### Eligibility criteria

Patients undergoing LSF at the Centre for Rheumatology and Spine Diseases, Rigshospitalet, Glostrup, from 1 October 2019 to 31 January 2021 fulfil the following criteria:

1. Low back pain ≥6 months
2. ≥ 18 years of age
3. Undergoing an instrumented posterolateral spinal fusion of 1–2 levels, with or without an intervertebral cage placed, performed from an anterior approach, posterior approach or from lateral access, for the degeneration of the lumbar spine. Degenerative conditions include disc degeneration, spinal stenosis with spondylolisthesis, and substantial spondylosis with or without spondylolisthesis [28]
4. Read and understand Danish
5. Live no more than 1.5 h of travel time by car from Rigshospitalet, Glostrup

#### Exclusion criteria

1. Previous LSF
2. One or more of following the conditions: infection, neoplasm, metastasis, metabolic bone disease, fractures, post-traumatic vertebral compression/deformity, other known autoimmune arthropathies
3. Diagnosed with a cognitive disorder (e.g. dementia, developmental disorders, or substance-induced cognitive impairment)
4. Other special conditions where a patient is judged to be unable to participate in the intervention by the surgeon or PI (HT) (fragile due to very high age, extremely poor functional level, psychiatric disease, or other serious comorbidities)

### Recruitment, screening, and enrolment

All spine surgeons at Rigshospitalet Glostrup will recruit patients to the trial. The surgeons will provide verbal and written information to the eligible patient at the preoperative consultation. If the patient accepts, the PI (HT) will contact the patient by telephone to check inclusion and exclusion criteria. If the patient is interested in participating in the trial, s/he will be invited to a meeting at the hospital with an independent assessor. The independent assessor will repeat information, and if the patient agrees to participate, s/he will sign an informed consent form. The independent assessor will hand out the baseline questionnaire and provide the patient with instructions on how to wear the accelerometer used to measure sedentary behaviour [29]. The meeting will be scheduled for the same day as the patient attends a pre-surgery back seminar to lessen burden and time spent by the patients.

### Intervention

**Fig. 2:**
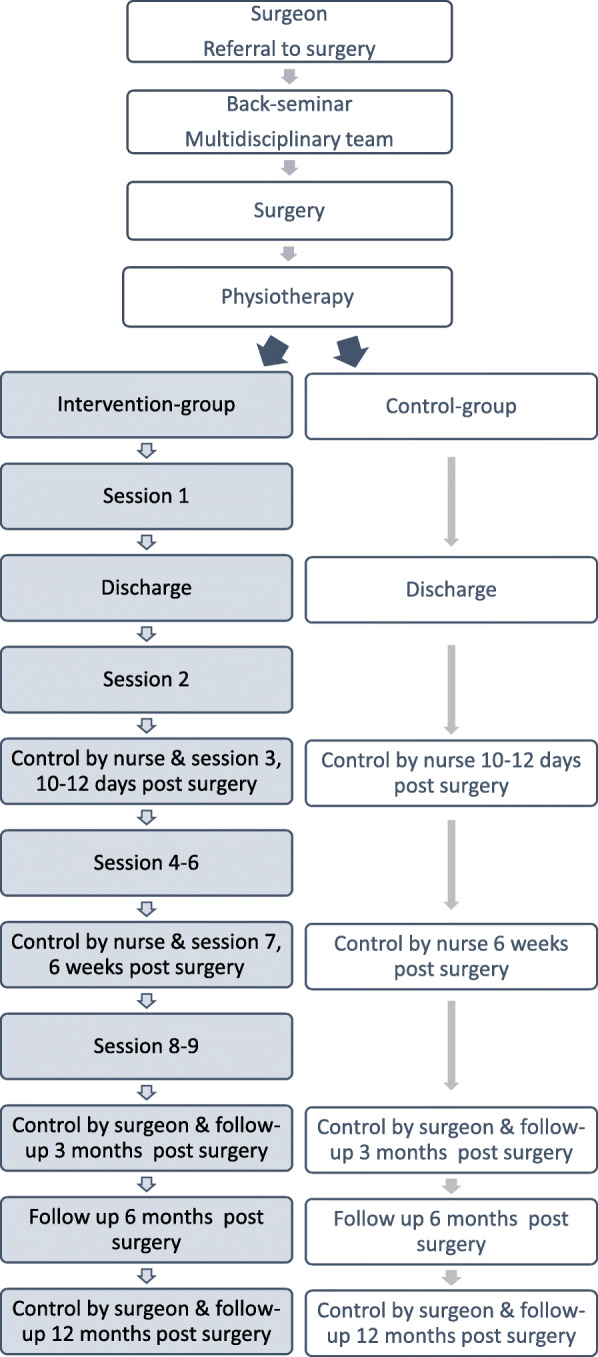
Overview of the two study groups, intervention group and control group

All patients (intervention and control) will receive usual care preoperatively and postoperatively from the Department of Rheumatology and Spine Diseases and the Department of Occupational Therapy and Physiotherapy, Rigshospitalet, Glostrup. An overview of the two groups is shown in Fig. 2. The description of GAPE and usual care in this protocol follows the TIDieRs checklist [30].

### Control group: usual care

#### Pre-surgery

Before surgery (1–2 weeks), the patient is invited to participate in a pre-surgery seminar, where s/he receives information about the time before, during, and after the LSF. The information covers anaesthesia, surgery, medication, mobilization, and how to use various aids. The seminar will be led by a nurse, a surgeon, an anesthesiologist, an occupational therapist, and a physiotherapist. The aim of the seminar is to gather the interdisciplinary team in one place for the patients and in this way give the best possible information regarding the surgery-procedure and the time just after surgery. The interdisciplinary team is blinded to the patient randomization and the content of GAPE.

#### During hospitalization

After surgery, the patient will be hospitalized for an average of 3–4 days. During hospitalization, a physiotherapist consults the patient one to three times to provide information and guidance on mobilization and instructions in a gradually progressing movement. The patient will have no restrictions on movement after surgery and is instructed by the physiotherapist to gradually return to their normal activity level. The patient will not receive any specific home exercise programme from the physiotherapist.

#### After discharge

Following discharge, the patient consults the nurse after 10–12 days at the outpatient clinic to remove stitches, talk about medication, and to answer any questions s/he might have regarding the period post-surgery. This will be repeated 6 weeks post-surgery.

Three months postoperatively, the patient will participate in a course of physical rehabilitation delivered by physiotherapists in a local community care centre. The post-surgical rehabilitation offered at the community care centres may vary in content and duration, although a typical course of post-surgical rehabilitation will contain an individual session with a physiotherapist followed by group training, in which the focus will be on stability, strength, and endurance of the back muscles. The patient will consult the surgeon at 3 and 12 months post-surgery, to discuss the surgical outcome and undergo a medical examination and an X-ray monitoring.

### Intervention: usual care supplemented by GAPE

In the intervention group, the patient will receive usual care, as described above, supplemented by GAPE distributed across nine sessions over a 10-week period. As described earlier, GAPE is based on a cognitive behavioural perspective. This builds on the assumption that human behaviour is effected by behavioural, cognitive, and affective factors, which include the patient’s perception of and response to pain [31].

**Fig. 3:**
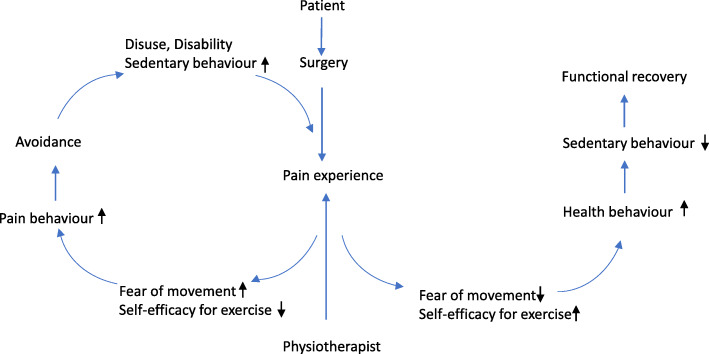
Modified version of the fear-avoidance model [33, 34]

The fear-avoidance model is one such cognitive behavioural model, recognized for understanding the development of chronic pain [32]. GAPE will use a modified version of the fear-avoidance model inspired by Woby et al. [33] and Lundberg [34], as shown in Fig. 3. The patients’ and physiotherapists’ former experiences, knowledge, and beliefs are factors influencing the patients’ experience of pain and thereby their self-efficacy for exercise and fear of movement.

### Components of GAPE

#### Graded activity

Graded activity is a technique based on the theory of operant conditioning and in the area of pain introduced by the American psychologist Wilbert Fordyce in 1976 [35]. The aim of the graded activity is to improve the patient’s functional ability by positive reinforcement of health behaviours and activity levels [36, 37]. By positive reinforcement means reinforcers such as attention, praise, rest from activities, and schedules which illustrate the occurrence of health behaviour. Graded activity has been tested in physiotherapy settings and has been found effective for the treatment of LBP [21, 38].

In GAPE, the role of the physiotherapist is to capture the patient’s thoughts and beliefs about pain and movement and the consequences of pain in the patient’s social life. The physiotherapist will capture this by an explorative interview (session 1) and observe the patient’s pain and/or health behaviours (sessions 1 and 2). Pain and health behaviours are the patient’s movement strategies despite the pain (e.g., duration of movement, way of moving, resting, breathing, grimacing) [39].

The patient will be asked to set three-five short-term goals for the next 10 weeks in close collaboration with the physiotherapist (session 2) [40]. The goals should involve physical activities that are specific, measurable, achievable, realistic, and time-targeted (SMART goals). The goals will be registered on the Patient-Specific Function Scale (PSFS) and will be the focal point for the activity/exercise programme which the physiotherapist plans for the patient [41, 42].

The patient’s current baseline tolerance level of each activity/exercise in the programme will be tested. Quotas will be set for the activities/exercises, balancing between the baseline tolerance level, the load of activities, and the knowledge provided through the interview and observation of the patient [35, 41]. Quotas will be lower than the baseline tolerance level, to secure a positive experience of performing the programme and to give the patient a sense of being in control. In subsequent sessions, quotas are systematically increased.

#### Pain education

The patient will have 1 session of pain education in GAPE (session 2) [43]. The underlying theoretical model for pain education is the modified fear-avoidance model (Fig. 3), as an approach to challenging the patient’s maladaptive pain cognition and to modify beliefs about movement despite pain [23, 44]. The pain education will target four overall questions:

1. What is pain?
2. Is my pain normal?
3. What can affect my pain?
4. What can I do to relieve my pain?

The pain education will be individually adapted to each patient, so the patient’s context and concerns regarding pain and movement are included. The educational material will be in the form of laminated worksheets. The educational material illustrates a simple explanation of the neurophysiology behind pain and suggestions of ways to manage pain in everyday life.

### The outlined intervention in GAPE

The outlined intervention will start just before discharge (session 1), and the final session (session 9) will take place 10 weeks postoperatively. The sessions will be held at the hospital, in the patient’s home and by telephone. The nine sessions are outlined in Additional file 2. Each session will be followed by home exercises matching the patient’s goals and quotas. The patient will be responsible for performing the programme until the following session is scheduled.

To improve adherence to the intervention, some of the sessions are placed on days where the patient is going to the usual check-up visit with the nurse at the hospital (sessions 3 and 7). Furthermore, three sessions (sessions 4, 6, and 8) will be of shorter duration and delivered by telephone.

Each intervention session will be registered by the physiotherapist as completed/not completed, to control for compliance to the scheduled intervention sessions.

### Physiotherapists’ training to deliver the intervention

Four physiotherapists from the Department of Occupational and Physiotherapy, Rigshospitalet, Glostrup, will perform the intervention (including the PI, i.e. HT). All physiotherapists managing the GAPE intervention have over 6 six years of experience in treating patients with complex LBP based on a cognitive behavioural perspective.

To attain skills in delivering graded activity, the physiotherapists have attended three training sessions with the PI (HT). During the sessions, theory and specific management approaches for the graded activity will be discussed and a training manual handed out [39]. Each physiotherapist will observe the PI (HT) perform a graded activity during an intervention period of one patient each (sessions 1 to 9). Furthermore, HT will supervise the three physiotherapists throughout the intervention period to ensure that the GAPE manual is followed.

To attain skills in delivering pain education, the physiotherapists will attend a course in pain mechanisms and pain education (“basic course in pain neuroscience”) [43]. The physiotherapists will also participate in a 1-day refresher course regarding pain theory and discuss and agree upon the exact pain education to be delivered in the intervention.

To ensure further treatment fidelity, all physiotherapists will be supervised by an experienced psychologist with expertise in the psychological treatment of chronic pain. Each physiotherapist will be supervised by the psychologist during one of their patient interviews (1st session). After 2 months, the psychologist will undertake a group supervision (lasting 3 h) again for intervention fidelity.

### Criteria for discontinuance

Patients allocated to the intervention group will be discontinued if he/she:

- Withdraws his/her consent
- Is scheduled for re-surgery
- Falls ill during the intervention period in such a way that it is not possible to continue the intervention.

### Outcomes and assessment

Data will be collected on four occasions during the trial: at baseline and at 3, 6, and 12 months post-surgery (see also Additional file 3). Patient-reported outcomes (PROs) will be completed at the hospital at baseline and at 3 and 12 months of follow-up, using an online data capture application (REDCap) [45, 46]. At 6 months of follow-up, the patient will receive an email with a link also via REDCap.

To prevent missing data, non-responders will be contacted by telephone shortly after data collection time points at 3, 6, and 12 months of follow-up, with a maximum of two reminders by email.

#### Primary outcome measure

The primary outcome is “reduction in sedentary behaviour” and will be measured at 3 months post-surgery. Sedentary behaviour will also be measured at 12 months of follow-up as a secondary outcome.

**Fig. 4:**
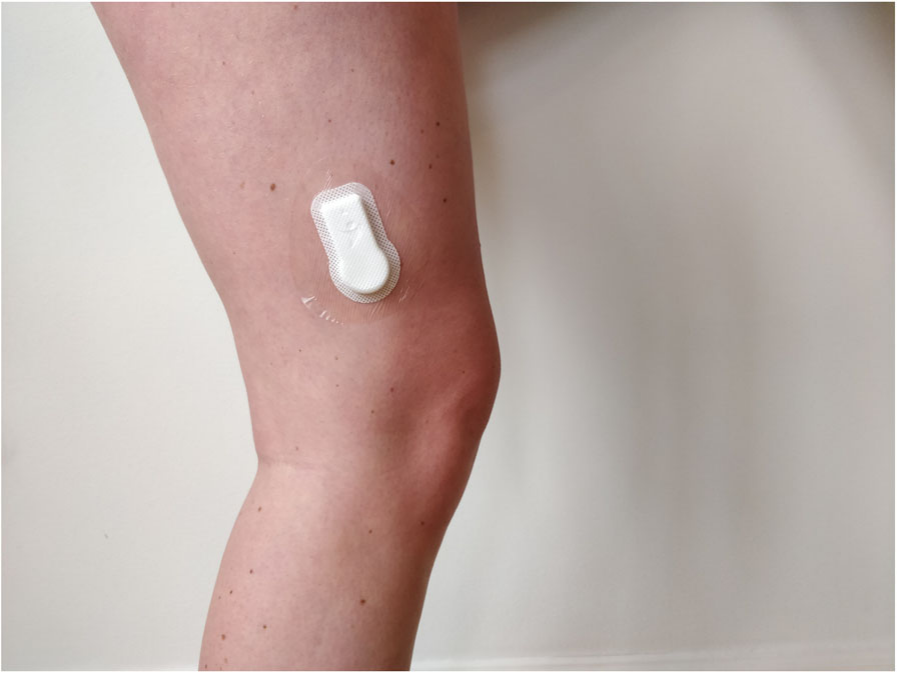
SENS accelerometer

Sedentary behaviour will be defined as: “any waking behaviour characterized by a sitting or reclining/lying posture” [47]. Sedentary behaviour will be assessed objectively as the number of minutes per day the patient is sedentary (lying down and sitting) measured with the SENS motion activity measurement system [29]. SENS is a small accelerometer placed within a small plaster to be worn discretely on the patient’s thigh (Fig. 4). The SENS motion system is considered a reliable and valid device for measuring sedentary behaviour [48]. The patients will wear the accelerometer for seven consecutive days during the week before surgery, and for 7 days at 3 and 12 months post-surgery.

#### Secondary outcome measures

Disability will be measured using the Oswestry Disability Index (ODI). ODI measures condition-specific disability and was developed for patients with LBP [49]. The Danish version of ODI has shown acceptable responsiveness, reliability, and validity [50, 51]. The ODI consists of ten items, covering pain intensity, personal care, lifting, walking, sitting, standing, sleeping, sex life, social life, and travelling. For each item, the patient chooses one of six answers, with 0 representing no difficulty in the activity and 5 representing maximal difficulty [50–52].

Pain in the back and legs is assessed using a visual analogue scale (VAS) with a possible score of 0 (“no pain”) to 100 (“the worst pain imaginable”) [53, 54]. The wording in the questionnaire is “Indicate your pain level for the past week by a mark on each line below, as shown in the example. The far-left side of the line corresponds to pain-free and the far-right corresponds to the worst possible pain. By placing a mark on the line you register how your pain has been within the past week”.

Fear of movement will be assessed using the 11-item short version of the Tampa Scale of Kinesiophobia (TSK-11). The TSK-11 has shown satisfactory validity, reliability, and responsiveness in a surgical spine population and in patients with chronic pain [9, 55]. Respondents are asked to indicate to what extent the items are a true description of the assumed association between movement and (re)injury on a 4-point Likert scale, ranging from strongly disagree to strongly agree.

Health-related quality of life will be assessed using the EuroQol 5 Dimensions three levels (EQ-5D-3L) [56]. EQ-5D-3L consists of the following five dimensions: mobility, self-care, usual activities, pain/discomfort, and anxiety/depression. Each dimension is divided into three levels: no problems, some problems, or extreme problems. EQ-5D-3L also includes a 20-cm vertical scale, where the respondent is asked to describe his/her own health with endpoints of “best imaginable health state” set at 100 and “worst imaginable health state” set at 0 [57]. The EQ-5D-3L has been validated in Danish, including the development of preference values [58] and Danish population norms [59].

“Self-efficacy for exercise” will be assessed using the questionnaire Self-efficacy for Exercise Scale (SEES). The SEES consists of nine items that measure how confident the patient is in doing exercise under different circumstances. The total range is from 0 to 90 points, where higher scores indicate a higher degree of self-efficacy for exercise. SEES has been found valid and reliable in a Scandinavian population [60].

#### Additional information

The following data will be retrieved from the patient’s (a) medical record and (b) a questionnaire developed for this trial.

Information from the patient’s electronic medical record will be as follows: sex, age, diagnosis and type of surgery (transforaminal lumbar interbody fusion (TLIF), posterior lumbar interbody fusion (PLIF), posterolateral fusion (PLF), oblique lateral interbody fusion (OLIF), lateral lumbar interbody fusion (LLIF), and anterior lumbar interbody fusion (ALIF). Comorbidity will be registered using the Charlson comorbidity index (CCI) [61, 62].

Information from the questionnaire will be as follows:

- Baseline data: height, weight, smoking, alcohol intake, employment, education status, and previous spine surgery.
- Satisfaction with the results after surgery: satisfaction will be used to evaluate the patient’s satisfaction regarding the achieved movement and pain after surgery. Patients will be asked to assess both their capacity to move their back and whether they feel safe moving their back on a VAS. The far-left side of the line corresponds to “very satisfied with my capacity to move my back post-surgery” and the far-right corresponds to “not at all satisfied with my capacity to move my back post-surgery”. Regarding pain, patients will be asked to compare back/leg pain before surgery until now. The patient can mark in five boxes, from “the pain has disappeared” to “the pain has worsened”. The patient will also be asked to evaluate the overall result of the operation, from “satisfied” to “not satisfied”.
- Adverse events: adverse events are defined as limitations in daily activities, sport activities, or work limitations, together with symptoms that cause patients to seek medical care. The events may be unrelated to the back (such as development or exacerbation of comorbidities), and the events are not necessarily causally linked to the LSF. The adverse events will be self-reported using a line with ample space for free text.
- Received guidance/training in physical exercise: to control for the received guidance/training in physical exercise during the time after surgery, both groups will be asked if they have received any guidance/training regarding physical activity, from whom, and the content, amount, and duration of the exercise.

### Randomization

**Fig. 5:**
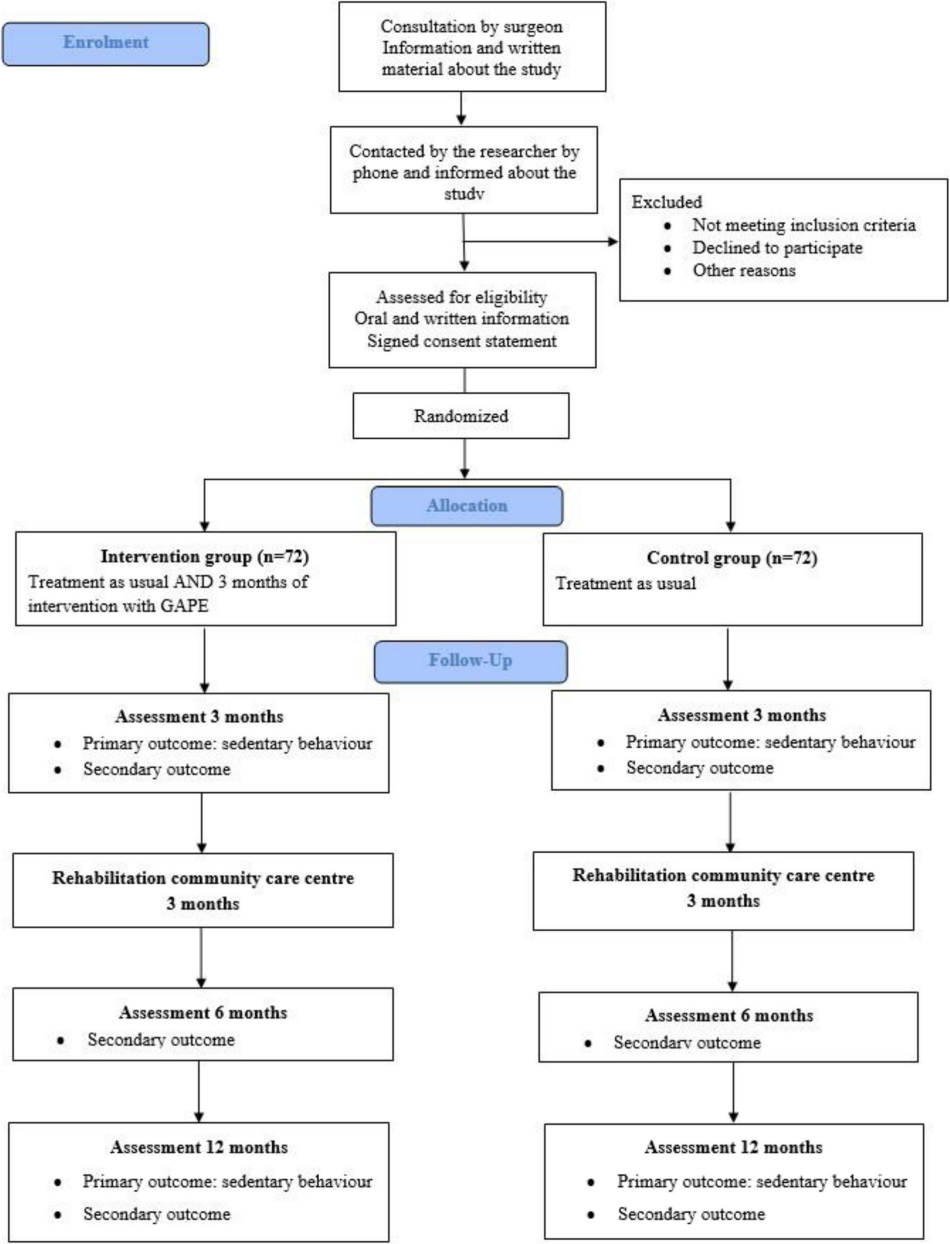
Flow diagram including participant timeline

Accordingly, 1 to 2 days after surgery, the patients will be allocated in a 1:1 ratio to either usual care (control group) or usual care together with GAPE (intervention group), taking into account the type of LSF (posterior versus anterior surgery approach) and smoking habits (i.e. block randomization). A co-investigator (MH) will be in charge of setting up the block randomization using SAS (SAS Institute Inc., Cary, NC, USA). The PI is blinded to the block randomization. The results of the randomization will be held in sealed opaque envelopes, until the intervention physiotherapists (including the PI) have delivered the envelopes to the included patients 1–2 days post-surgery (see Fig. 5, flow diagram).

### Allocation and blinding

The patient will receive the allocation number just before discharge, so health professionals administering the usual postoperative care during hospitalization (surgeons, nurses, occupational therapists, and physiotherapists) will be blinded to randomization. The group allocation numbers will be delivered in sealed envelopes by HT.

Two blinded assessors (physiotherapists) will collect informed consents and will be responsible for the entire outcome assessment (at baseline pre-surgery and at 3, 6, and 12 months of follow-up). These assessors will be trained by HT in requirements of the trial and the standardized measurement procedures, including a manual for the procedures in the trial.

Due to the nature of the intervention, neither the patients, the intervention physiotherapists, nor the PI will be blinded to the group allocation.

### Analyses

#### Sample size

The power calculation is based on the primary outcome, sedentary behaviour. A mean difference of 60 min per day is considered a clinically significant difference, with a standard deviation of 115 [63]. A sample size of 59 per group is required to obtain a power of at least 80% to detect the mean difference of 60 min. To account for a 20% drop-out, we will include 144 patients in total.

### General statistical approach

A detailed statistical analysis plan (SAP) will be produced before the enrolment of the last patient.

Assessments of outcomes and construction of confidence intervals (CIs) for continuous measures will be based on an analysis of covariance (ANCOVA; including group as the main factor and baseline measure as a covariate).

Superiority will be claimed if the computed 95% CI of the estimated group difference in the time spent in a sedentary position does not include 0 in the intention-to-treat (ITT) population.

All statistical tests will be two-sided and statistical significance will be claimed if the computed *p* value is equal to or less than 0.05.

### Analysis of population sets

For the assessment of superiority, we will use the ITT population in the primary analysis, as it is the most conservative approach.

### Study population definitions

#### ITT population

This consists of all randomized patients in the groups to which they were randomly assigned, regardless of the treatment they actually receive.

#### Per-protocol (PP) population

All patients adhere to the planned protocol, defined by the following criteria for the two groups:

The intervention-group (GAPE) has the following:

- A baseline measurement
- At least one post-baseline measurement (3, 6, or 12 months post-surgery)
- Attended the first two sessions at the hospital and in the patient’s home .(respectively sessions 1 and 2)
- Attended at least two of the scheduled graded activity appointments; this does not include telephone sessions (a GAPE intervention attendance record will be used for documentation)

The control group (usual care) has the following:

- A baseline measurement
- At least one post-baseline measurement (3, 6, or 12 months post-surgery)
- Has not received other kinds of active cognitive behavioural treatment during the first 3 months post-surgery

### Data management

The blinded assessor will give each participant a trial identification number, and all data will be de-identified. The identification list with participant information and trial study number will be kept in REDCap. HT and MH will have access to the final dataset in REDCap. The signed consent will be kept in locked filing cabinets behind double-locked doors.

### Risks, side effects, and inconvenience

Participation in the trial is expected to be associated with a minimal risk of side effects. The project is planned so that hospital visits should disturb the patients as little as possible. The patients will have to visit Rigshospitalet, Glostrup, 1–2 extra times, and documented travel expenses will be refunded on request [64].

Former studies of early post-surgical rehabilitation following LSF have not reported any risks or side effects [18]. In addition, co-investigator, spine surgeon RBA, has assessed the intervention description for any possible risks regarding the fusion material and does not consider that the GAPE intervention entails an increased risk of stress on the pedicle screw fixation system. In the event that any patient shows signs of new neurological symptoms, such as paresis, unexpected worsening of preoperative lumbar or radicular pain or sensitivity changes, or unexpected changes in the surgery wound during the intervention period, the physiotherapist will contact the surgeon.

Regarding the SENS accelerometer, this might entail some discomfort, when the accelerometer is removed, similar in magnitude to taking off normal plaster. Furthermore, there is a small risk of an allergic reaction to the plaster holding the SENS accelerometer. Patients will receive specific skin care advice if this should happen.

Patients are covered by the “patient-compensation agreement”, if anything unexpected should happen during the intervention.

Patients included in the project cannot participate in other kinds of research projects with active interventions or receive other kinds of active cognitive behavioural treatment during the first 3 months post-surgery. This is to ensure that the intervention and the control group are comparable and only differ by the GAPE intervention being evaluated.

### Patient involvement in the study

Prior to the development of the GAPE intervention, HT conducted semi-structured informal interviews with five volunteer patients—both immediately after LSF and 1 month postoperatively. The interviews were performed to include the patients’ perspective of pain, movement, and thoughts after LSF and to incorporate their ideas of an ideal early postoperative intervention.

To ensure that the patient’s perspective remains a vital part of this project, a patient operated in 2015 with LSF has agreed to participate as a patient research partner (PRP) throughout the entire project period. The PRP has read, commented, on and approved the participant information. Furthermore, on several occasions, the PRP has commented on drafts of the intervention and the choice of outcomes.

The outlined intervention has also been pilot tested by HT on four patients with LSF.

### Dissemination

The outlined project is expected to produce three scientific articles in internationally peer-reviewed journals, whether the results be positive, negative, or inconclusive. Additionally, the results of the project will be communicated in both academic and public fora.

## Discussion

Research in the field of post-surgical rehabilitation after LSF calls for further examination of early active approaches, incorporating a truly bio-psycho-social focus [18, 19]. This trial is based on the assumption that behavioural, cognitive, and affective factors each contribute to human behaviour and amplify and interact with physical pathology [31]. In GAPE, the patient’s home is included as a location for the intervention, where s/he is interviewed and observed, which informs the guideline for the early post-surgical rehabilitation. Previous research in the field of rehabilitation after LSF [18, 19] has not investigated such an individual approach, and we hypothesize that it will contribute to improved health behaviour and thereby faster functional recovery after LSF.

Graded activity is a technique originally used by psychologists [65], and it can be questioned whether physiotherapists can manage this kind of intervention. In this trial, the physiotherapists have experience and competences in communicating about pain and treating patients from a cognitive behavioural perspective, and it is thereby hypothesized that they are capable of performing GAPE. This is supported by a systematic review by Bruner et al. [66] which finds that operant conditioning can be integrated into an ambulant physiotherapy setting. The previously mentioned study by Archer et al. also confirms this by finding positive effects of a cognitive behavioural approach applied by a physiotherapist to patients undergoing LSF [19].

Previous investigations in post-surgical rehabilitation after LSF have mainly used outcomes based on PROMs. Because the purpose of the current intervention is to change the patient’s beliefs about movement despite pain, and thereby reduce pain-induced sedentary behaviour, the performance-based outcome “sedentary behaviour” is considered a relevant primary outcome. Furthermore, accelerometers are recommended over self-reports in terms of measuring sedentary behaviour, because they are not influenced by recall bias, overestimations and social desirability [67, 68]. The study will thereby use both performance-based and patient-reported outcomes, and from our perspective, this will give a more comprehensive picture of the patient.

The strength and quality of the study lie in its thorough preparation. Previous patients with LSF have been involved in the preparation phase by way of interviews, the involvement of a PRP, and by way of a pilot test. Furthermore, to consolidate the quality of the outlined intervention throughout the intervention period, the physiotherapists are trained and supervised before and during the intervention period.

In this intervention, we hope to capture the specific preferences for the post-surgical rehabilitation of each patient and, in addition, include the patient’s environment. We hope to inspire other clinical settings (hospitals, private clinics, and community care centres) to view post-surgical rehabilitation in a broader perspective and discuss how the patient’s own preferences and experiences of pain and movement can be captured and used in a rehabilitation setting, regardless of diagnosis.

### Trial status

**Table:** 

Protocol version (date)	10 (25 March 2020)
Date of the first enrolment	1 October 2019
Completed recruitment	31 January 2021
Recruitment status	Recruiting

## Supplementary information

**Additional file 1:.** SPIRIT 2013 Checklist: Recommended items to address in a trial protocol and related documents.

**Additional file 2:.** The outlined intervention.

**Additional file 3:.** Outcomes and assessment period.

## Data Availability

Not applicable.

## Abbreviations

ALIF: Anterior lumbar interbody fusion
CLBP: Chronic low back pain
CIs: Confidence intervals
GAPE: Graded activity and pain education
HT: Heidi Tegner
ITT: Intention-to-treat
LLIF: Lateral lumbar interbody fusion
LBP: Low back pain
LSF: Lumbar spinal fusion
MRH: Marius Henriksen
OLIF: Oblique lateral interbody fusion
ODI: Oswestry Disability Index
PRP: Patient research partner
PROMs: Patient-reported outcome measures
PI: Principal investigator
EQ-5D: European Quality of Life-5 Dimensions
RCT: Randomized controlled trial
RBA: Rachid Bech-Azeddine
SEES: Self-efficacy for Exercise Scale
TSK-11: Short version of Tampa Scale of Kinesiophobia
SPIRIT: Standard Protocol Items: Recommendations for Interventional Trials
MCID: Minimal clinically important difference
TLIF: Transforaminal lumbar interbody fusion
VAS: Visual analogue scale
