# Supplementary material for: The effect of graded activity and pain education (GAPE): an early post-surgical rehabilitation programme after lumbar spinal fusion—study protocol for a randomized controlled trial
Source: Trials. 2020 Sep 15;21:791. doi: 10.1186/s13063-020-04719-y (PMC7493936; doi:10.1186/s13063-020-04719-y)
Supplement: Supplementary file 2 — Additional file 2:. The outlined intervention. [file 13063_2020_4719_MOESM2_ESM.docx]

File 2. Description of content of the GAPE intervention (graded activity and pain education)

| **Session no. Setting (days post-surgery)** | **Aim** | **Technique** |
| --- | --- | --- |
| 1. Hospital  (2 days post-surgery) | Explore the patient’s beliefs and thoughts about pain and movement | Explorative interview |
|  | Identify the patient’s pain/health behaviour | Observation of the patient’s movement strategies post-surgery |
| 2. Patient’s home  (5-7 days post-surgery) | Explore the patient’s beliefs and thoughts about pain and movement | Explorative interview |
|  | Identify the patient’s pain/health behaviour | Observation of the patient’s movement strategies post-surgery |
|  | Provide the patient with an understanding of the complexity of pain and the association between pain behaviour, physical activity and pain | Pain education guided by information from interviews and observation |
|  | Form an individualized plan for functional recovery | Formulate 3-5 short-term functional goals with GAPE on PSFS |
| 3. Hospital  (10-12 days post-surgery) | Challenge the patient’s feelings and thoughts regarding movement of the back and physical activity | Introduce the patient to activity/exercise-programme based on graded activity and the 3 short-term functional goals  Set realistic quotas for the programme by testing baseline tolerance level and set quota below this level  Positive reinforcement |
| 4. Telephone  (15-18 days post-surgery) | Challenge beliefs about movement and self-efficacy for exercise | Secure level for graded activity by checking quota level and adjust if necessary |
|  | Maintain the patient’s motivation for GAPE | Positive reinforcement of health behaviours |
| 5. Patient’s home  (20-25 days post-surgery) | Challenge beliefs about movement and self-efficacy for exercise | Include pain education if suitable  Follow up on activity/exercise-programme  Quota adjustment  If goals are reached new goals are set |
|  | Increase health behaviour | Positive reinforcement of health behaviours |
| 6. Telephone  (30-35 days post-surgery) | Challenge beliefs about movement and self-efficacy for exercise | Secure level for graded activity by checking quota level and adjust if necessary |
|  | Maintain the patient’s motivation for GAPE | Positive reinforcement of health behaviours |
| 7. Hospital  (40-45 days post-surgery) | Challenge beliefs about movement and self-efficacy for exercise | Include pain education if suitable  Follow up on activity/exercise-programme  Quota adjustment  If goals are reached new goals are set |
|  | Increase health behaviour | Positive reinforcement of health behaviours |
| 8. Telephone  (55-60 days post-surgery) | Challenge beliefs about movement and self-efficacy for exercise | Secure level for graded activity by checking quota level and adjust if necessary |
|  | Maintain the patient’s motivation for GAPE | Positive reinforcement of health behaviours |
| 9. Hospital (70 days post-surgery) | Challenge beliefs about movement and self-efficacy for exercise | Include pain education if suitable  Follow up on activity/exercise-programme  Quota adjustment  Goal setting for the time after intervention |
|  | Increase health behaviour | Positive reinforcement of health behaviours |
