# Supplementary material for: The effect of graded activity and pain education (GAPE): an early post-surgical rehabilitation programme after lumbar spinal fusion—study protocol for a randomized controlled trial
Source: Trials. 2020 Sep 15;21:791. doi: 10.1186/s13063-020-04719-y (PMC7493936; doi:10.1186/s13063-020-04719-y)
Supplement: Supplementary file 3 — Additional file 3:. Outcomes and assessment period. [file 13063_2020_4719_MOESM3_ESM.docx]

File 3. Outcomes and assessment period

| **Variable** | **Objective measurement** | **Patient-reported outcome measures (PROM)** | **Baseline**  **(1 week pre-**  **surgery)** | **3 months post-**  **surgery** | **6 months post-**  **surgery** | **1-year post-**  **surgery** |
| --- | --- | --- | --- | --- | --- | --- |
| Baseline information | | | | | | |
| Age |  | Questionnaire | X |  |  |  |
| Gender |  | Questionnaire | X |  |  |  |
| Height |  | Questionnaire | X |  |  |  |
| Weight |  | Questionnaire | X |  |  |  |
| Employment |  | Questionnaire | X |  |  |  |
| Smoking |  | Questionnaire | X |  |  |  |
| Duration of symptoms |  | Questionnaire | X |  |  |  |
| Other back surgeries |  | Questionnaire | X |  |  |  |
| Comorbidities |  | Charlson Comorbidity Index | X |  |  |  |
| Educational level |  | Questionnaire | X |  |  |  |
| Diagnosis | Medical record |  | X |  |  |  |
| Type of surgery | Medical record |  | X |  |  |  |
| Primary outcome | | | | | | |
| Sedentary  Behaviour | Accelerometer (SENS) |  | X | X |  | X |
| Secondary outcomes | | | | | | |
| Disability |  | Ostwestry Disability Index (ODI)  Questionnaire | X | X | X | X |
| Pain |  | Visual Analogue Scale (VAS)  Questionnaire | X | X | X | X |
| Fear of movement |  | Short version of Tampa Scale of Kinesiophobia (TSK-11)  Questionnaire | X | X | X | X |
| Confidence in exercise |  | Self-efficacy for exercise Scale (SEES)  Questionnaire | X | X | X | X |
| Quality of life |  | Quality of life survey (EQ-5D)  Questionnaire | X | X | X | X |
| Patient satisfaction with results of the surgery |  | Questionnaire |  | X | X | X |
